# Carotenoid Extract Derived from *Euglena gracilis* Overcomes Lipopolysaccharide-Induced Neuroinflammation in Microglia: Role of NF-κB and Nrf2 Signaling Pathways

**DOI:** 10.1007/s12035-021-02353-6

**Published:** 2021-03-21

**Authors:** Anna Piovan, Raffaella Filippini, Giovanni Corbioli, Vanessa Dalla Costa, Elisabetta Maria Vittoria Giunco, Gianluca Burbello, Andrea Pagetta, Pietro Giusti, Morena Zusso

**Affiliations:** Department of Pharmaceutical and Pharmacological Sciences, University of Padua, 35131 Padua, Italy

**Keywords:** Neuroinflammation, Microglia; *Euglena gracilis*, Pro-inflammatory cytokines, Nuclear factor-κB, Nuclear factor erythroid 2–related factor 2

## Abstract

Activation of microglia results in the increased production and release of a series of inflammatory and neurotoxic mediators, which play essential roles in structural and functional neuronal damage and in the development and progression of a number of neurodegenerative diseases. The microalga *Euglena gracilis* (Euglena), rich in vitamins, minerals, and other nutrients, has gained increasing attention due to its antimicrobial, anti-viral, antitumor, and anti-inflammatory activities. In particular, anti-inflammatory properties of Euglena could exert neuroprotective functions in different neurodegenerative diseases related to inflammation. However, the mechanisms underlying the anti-inflammatory effect of Euglena are not fully understood. In this study, we investigated whether Euglena could attenuate microglia activation and we also studied the mechanism of its anti-inflammatory activity. Our results showed that non-cytotoxic concentrations of a Euglena acetone extract (EAE) downregulated the mRNA expression levels and release of pro-inflammatory mediators, including NO, IL-1β, and TNF-α in LPS-stimulated microglia. EAE also significantly blocked the LPS-induced nuclear translocation of NF-κB p65 subunit and increased the mRNA expression of nuclear factor erythroid 2–related factor (Nrf2) and heme oxygenase-1 (HO-1). Furthermore, the release of pro-inflammatory mediators and NF-κB activation were also blocked by EAE in the presence of ML385, a specific Nrf2 inhibitor. Together, these results show that EAE overcomes LPS-induced microglia pro-inflammatory responses through downregulation of NF-κB and activation of Nrf2 signaling pathways, although the two pathways seem to get involved in an independent manner.

## Introduction

In the central nervous system (CNS), chronic inflammatory processes are closely linked to neurodegenerative diseases. Key regulators of CNS inflammatory response are microglial cells, the major cellular components of the CNS innate immune system. Microglia are dual-functioning cells that play complex and crucial functions in the normal as well as in the diseased CNS [1, 2]. In response to CNS damage, microglia become activated and orchestrate the inflammatory response by releasing inflammatory factors, with the ultimate aim of healing the damaged tissue. However, if the activation process becomes dysregulated, over-activated microglia produce high levels of pro-inflammatory and cytotoxic mediators (e.g., reactive oxygen species (ROS), nitric oxide (NO), prostaglandin E_2_, interleukin (IL)-6, IL-1β, tumor necrosis factor-α (TNF-α), etc.), which contribute to synaptic dysfunction and neuronal cell death [3, 4]. Crucial to microglia activation is the production of ROS and NO, which can activate kinase cascades and several transcription factors, such as nuclear factor-κB (NF-κB), which in turn regulates the expression of hundreds of inflammatory genes, resulting in a vicious cycle that increases the neuroinflammatory process and subsequent neurodegeneration [5]. Controlled ROS levels are maintained by the nuclear factor erythroid 2–related factor 2 (Nrf2) and its downstream enzymes, such as heme oxygenase 1 (HO-1). Nrf2 is a key factor of endogenous defense system, that activates antioxidant and cytoprotective genes in response to oxidative stress. Furthermore, Nrf2 modulates activation of immune cells, including microglia, and its deletion has been associated with increased sensibility to neuroinflammation induced by lipopolysaccharide (LPS) [6, 7]. Thus, therapeutic strategies directed at controlling microglia activation and the excessive production of pro-inflammatory and pro-oxidant factors, by targeting NF-κB and/or Nrf2, could be relevant in the context of inflammation-mediated neurodegeneration.

Proper nutrition is a key component of a healthy lifestyle and certain dietary compounds seem to play a crucial role in the prevention of neurodegenerative diseases, suggesting that dietary interventions may represent useful tools for preventing neurodegeneration [8]. A healthy diet contributes to the physiological development of the CNS and participates in the maintenance of neuronal plasticity [9]. For example, a balanced diet rich in bioactive compounds can reduce the risk of cognitive decline and dementia [10, 11]. On the contrary, an unbalanced diet and obesity have a severe impact on brain functions and are associated with increased oxidative stress, mitochondrial dysfunction, amyloid precursor protein expression, neuroinflammation, and apoptosis, all crucial events implicated in the onset and/or progression of neurodegenerative diseases [12, 13]. Functional foods, dietary supplements, and nutraceuticals have recently become increasingly attractive as therapeutic interventions in the management of neurodegenerative disease, particularly Alzheimer’s disease. In this context, microalgae represent promising opportunities in the field of functional foods due to their high content of a mixture of bioactive molecules, which major compounds are vitamins (e.g., B12), polysaccharides, polyunsaturated fatty acids (e.g., ω-3 and ω-6 polyunsaturated fatty acids), minerals, enzymes, essential amino acids (e.g., leucine, isoleucine, and valine), fibers, and pigments (e.g., chlorophylls and carotenoids) [14–16]. In particular, carotenoids, which are widely distributed in nature, are the most interesting compounds. Usually, this family of pigments is divided into molecules with hydrocarbon structures, generally named carotenes, and molecules which also contain oxygen atoms, called xanthophylls [17]. Carotenoids play a variety of important functions and have been considered compounds able to fight the free radicals, reduce the risk of cancer, and prevent cardiovascular and neurodegenerative diseases, among others [18–20]. The multiple health benefits of carotenoids are supposed to be due to their antioxidant and anti-inflammatory functions, considering that carotenoids can sequestrate free radicals released in the human body under stress conditions, as well as reduce inflammatory mediators [21–23].

Among the microalgae, the flagellate microalga *Euglena gracilis* (Euglena) has emerged as an interesting nutritional and functional supplement, as it is an excellent source of dietary protein, pro-vitamins, lipids, and the β-1,3-glucan paramylon, uniquely produced by euglenoids [24]. The whole dried powder and/or water or organic solvent extracts of different Euglena species have several pharmacological properties, such as antimicrobial, anti-viral, antitumor, and antioxidant activities [25–27]. In addition, supplementation with Euglena supports immune functions [28].

Based on this evidence, the aim of the present study was to explore whether an acetone carotenoid-rich extract from the microalga Euglena possesses anti-inflammatory effects regulating microglia activation. Then, the potential mechanism that regulates the observed effects was also clarified. We found that the Euglena acetone extract (EAE) modulated microglial activation by inhibiting the expression and release of pro-inflammatory molecules in LPS-induced neuroinflammation through a mechanism that involves NF-κB and Nrf2 signaling pathways.

## Materials and Methods

### Reagents

Unless otherwise specified, all reagents were from Sigma-Aldrich (Milan, Italy). Tissue culture media, antibiotics, and fetal bovine serum (FBS) were obtained from Life Technologies (San Giuliano Milanese, Italy). LPS (Ultra-Pure LPS-EB from *Escherichia coli*, 0111:B4 strain) was purchased from InvivoGen (InvivoGen Europe, Toulouse, France). Primary antibodies included: rabbit anti-ionized calcium-binding adaptor molecule 1 (Iba1, 019-19741, Wako Pure Chemical Industries, Ltd., Osaka, Japan), mouse anti-p65 (NF-κB p65, sc-8008, Santa Cruz Biotechnology, Santa Cruz, CA, USA), mouse anti-NOS2 (sc-7271, Santa Cruz Biotechnology). Alexa Fluor 488 and 555 secondary antibodies were from Invitrogen (A11008 and A21422, Milan, Italy). Enzyme-linked immunosorbent assay (ELISA) kits were obtained from Antigenix America (Huntington Station, NY, USA). Falcon tissue culture plasticwares were purchased from BD Biosciences (SACCO srl, Cadorago (CO), Italy).

### *Euglena gracilis* Cultures

Euglena strain (1224-5/27) was obtained from the Culture Collection of Algae (SAG, Göettingen, Germany). Cells were cultured in CM liquid medium supplemented with 3 g/L sodium acetate, in Erlenmeyer flasks [29]. The pH was adjusted to 6.8. Cultures were maintained at 24–26 °C under a photoperiod (16/8-h light-dark cycle). A growth curve was calculated by determination of the fresh weight every second or third day over a cultivation period of 14 days. As soon as the cultures reached the stationary growth phase, an inoculum was transferred in fresh medium, whereas the remaining algal cells were harvested by centrifugation and concentrated to a biomass slurry that was washed twice with deionized water. Finally, the cleaned samples were stored at − 20 °C until use.

### Preparation and Analysis of *Euglena gracilis* Extract

Fresh cells were extracted with acetone in an ultrasonic bath for 20 min at room temperature and then centrifuged at 4400 rpm for 10 min at 4°C. The extraction process was repeated three times and the combined acetone extracts were concentrated under reduced pressure in a rotary evaporator. The concentrated solution was then lyophilized. Finally, the extract was stored at 4 °C until use.

The content of pigments (chlorophyll a, chlorophyll b, and carotenoids) in the acetone extract was determined as previously described by Yang et al. [30]. One aliquot of the extract was solubilized with acetone:water (4:1) and, after proper dilution, the maximum absorbance was read at 663 nm, 646 nm, and 470 nm for chlorophyll a, chlorophyll b, and total carotenoids, respectively. The content of pigments was then calculated using the following equations:

$$ {\displaystyle \begin{array}{l}\mathrm{Chlorophyll}\ \mathrm{a}\ \left(\upmu \mathrm{g}/\mathrm{mL}\right)=12.25{\mathrm{A}}_{663}\hbox{-} 2.25\ {\mathrm{A}}_{646}\\ {}\mathrm{Chlorophyll}\ \mathrm{b}\ \left(\upmu \mathrm{g}/\mathrm{mL}\right)=20.31{\mathrm{A}}_{646}\hbox{-} 2.25\ {\mathrm{A}}_{663}\\ {}\mathrm{Total}\ \mathrm{carotenoids}\ \left(\upmu \mathrm{g}/\mathrm{mL}\right)=\left(1000\ {\mathrm{A}}_{470}\hbox{-} 2.27\ \mathrm{Chlorophyll}\ \mathrm{a}\hbox{-} 81.4\ \mathrm{Chlorophyll}\ \mathrm{b}\right)/227\end{array}} $$

Results were expressed as mg/g of dry weight of extract.

The carotenoid analysis of EAE was performed using an Agilent 1100 HPLC Series System (Agilent, Santa Clara, CA, USA) equipped with degasser, quaternary gradient pump, column thermostat, and UV-Vis detector. A Gemini 5-μm C6-Phenyl column (250 × 4.6 mm) from Phenomenex (Torrance, CA, USA) was employed, at 40 °C. Analyses were done in the isocratic mode, using acetonitrile:methanol (10:90; v/v) at a flow rate of 1 mL min^−1^, with an injection volume of 10 μL; detection was at 280, 365, and 460 nm. Carotenoid content was expressed as β-carotene equivalents.

### Cell Cultures

Animal-related procedures were performed in accordance with EU Directive (2010/63/EU) for animal experiments and those of the Italian Ministry of Health (D.L. 26/2014) and were approved by the Institutional Review Board for Animal Research (Organismo Preposto al Benessere Animale, OPBA) of the University of Padua and by the Italian Ministry of Health (protocol number 958/2016-PR). Animal procedures were carried out in compliance with the ARRIVE guidelines [31, 32]. Animals were maintained under controlled conditions (22-24°C, 50%-60% humidity), with free access to water and food on a 12-h light/dark cycle (lights on at 7:00 am). One-day-old Sprague-Dawley rat pups (CD strain) of both sexes were rapidly decapitated, minimizing suffering, discomfort, or stress. Primary microglial cells were isolated from mixed glial cell cultures prepared from the cerebral cortex, as previously described [33], with slight modifications. Briefly, when mixed glial cultures reached confluence (typically 7–10 days after isolation), microglia were separated from the astroglial monolayer by shaking (200 rpm for 1 h at 37 °C), re-suspended in high-glucose Dulbecco’s modified eagle medium (DMEM) supplemented with 2 mM L-glutamine, 10% heat-inactivated FBS, 100 units/mL penicillin, 100 μg/mL streptomycin and 50 μg/mL gentamicin (growth medium), and plated on poly-l-lysine-coated (10 μg/mL) plastic wells at a density of 1.50 × 10^5^ cells/cm^2^. Cells were allowed to adhere for 45 min and then washed to remove non-adhering cells. Cultures obtained using the shaking procedure generated 97% microglia immunopositive to a primary polyclonal antibody against Iba1 (1:800), a marker for microglia cell types [34, 35]. Cells were maintained at 37 °C in a humidified atmosphere containing 5% CO_2_/95% air. EAE was suspended in dimethylsulfoxide (DMSO) just before use and added to the cultures so as not to exceed 0.1% of the total volume. Control cultures contained the same concentration of DMSO.

### Cell Viability Assay

Microglial cell viability was evaluated by a colorimetric method utilizing the protein-binding dye sulforhodamine B (SRB) [36, 37]. Cells were seeded in poly-l-lysine coated 96-well plates (50,000 cells/well) in growth medium and allowed to adhere overnight. Growth medium was replaced with serum-free medium 2 h before treatment for 16 h with increasing concentrations of EAE, in the absence or presence of 100 ng/mL Ultra-Pure LPS-EB. After the incubation, the medium was replaced with cold 10% trichloroacetic acid, and plates were incubated for 1 h at 4 °C. Following this fixation step, cells were stained with 0.4% SRB and left at room temperature for 30 min. The bound protein stain was solubilized with 10 mM Tris base. The absorbance was then measured at 570 nm in a microplate reader. Absorbance of vehicle-treated cultures was considered as 100% cell viability.

### Nitric Oxide Assay

Primary microglia were pretreated for 1 h with increasing concentrations of EAE and then stimulated with 100 ng/mL Ultra-Pure LPS-EB for an additional 16 h. Thereafter, the production of NO was determined by the indirect measurement of its stable oxidized metabolites nitrites, using the Griess reaction. Fifty μL of the cell culture medium and an equal volume of the Griess reagent were mixed, incubated at room temperature for 15 min, and the absorbance at 540 nm was measured in a microplate reader. The nitrite concentration in the supernatant was quantified using a standard curve of sodium nitrite.

### Cytokine Determination

Primary microglia were pretreated for 1 h with increasing concentrations of EAE and then stimulated with Ultra-Pure LPS-EB for an additional 16 h. At the end of incubation, culture medium was collected and IL-1β and TNF-α assayed using commercially available ELISA kits, according to the manufacturer’s instructions. Cytokine concentrations (pg/mL) in the medium were determined by reference to standard curves obtained with known amounts of IL-1β or TNF-α.

### Immunofluorescence and Image Analysis

Microglia were grown on coverslips in 24-well plates, pretreated for 1 h with the EAE, and then stimulated with 100 ng/mL Ultra-Pure LPS-EB for an additional 90 min or 16 h for the analysis of NF-κB activation or iNOS expression, respectively. Cells were fixed with 4% paraformaldehyde (pH 7.4, for 15 min at room temperature) and subsequently non-specific staining was blocked by incubating with 5% normal goat serum/0.1% Triton X-100 in PBS for 1 h at room temperature. Cells were then incubated sequentially with primary (2 h) and secondary antibodies (1 h) in the above blocking solution. The antibodies used were rabbit anti-Iba1 (1:800), mouse anti-p65 (NF-κB p65, 1:500), and mouse anti- iNOS (NOS2, 1:500) primary antibodies, followed by the Alexa Fluor 488- or 555-conjugated secondary antibodies (1:1000). Cells were thoroughly washed between steps with PBS. Immunostaining control included omission of the primary antibody. Nuclei were stained with 4,6-diamidino-2-phenylindole (DAPI; 0.1 μg/mL) and coverslips were mounted on microscope slides with Fluoromount-G mounting medium (Fisher Scientific, Milan, Italy) [38]. Fluorescent images were captured with a confocal laser-scanning microscope (Zeiss LSM 800; Carl Zeiss AG, Germany) and microscope settings were kept constant for all images. For each image, three z-stacks (50-μm optical section, 1.5-μm total Z-span) were acquired with a 63x, NA 1.4, oil-immersion objective. All images were taken considering the middle of nuclei as the central plane for z-stack. ImageJ software (National Institutes of Health, Bethesda, MD, USA) was used to flatten each z-stack image into a single image, representing the sum of the contributes from each focal plane. Then, the fluorescence emission intensity of single cells was profiled using ImageJ software. To quantitatively evaluate subcellular distribution of the p65 subunit, the relative staining intensities in the nucleus and cytoplasm were monitored from five random fields for each condition from three independent experiments. Cytoplasmic and nuclear fluorescence intensities were calculated using ImageJ software and are expressed as a percentage of nuclear and cytoplasmic staining.

### Real-time Polymerase Chain Reaction

Primary microglia were pretreated for 1 h with the acetone extract and then stimulated with 100 ng/mL Ultra-Pure LPS-EB for an additional 6 h. At the end of incubation, total RNA was extracted from cells by QIAzol (Invitrogen), according to the manufacturer’s instructions. RNA integrity and quantity were determined by RNA 6000 Nano assay in an Agilent BioAnalyser (Thermo Scientific, Milan, Italy). Reverse transcription was performed with Superscript III reverse transcriptase (Invitrogen). The real-time PCR reaction was performed as described previously [39]. Primer sequences are listed in Table 1. The expression of target genes *HO-1*, *IL-1β*, *iNOS*, *Gas6*, *Nrf2*, and *TNF-α* was normalized to the expression of the housekeeping gene β-actin, used as reference. The relative expression levels were quantified using the Pfaffl method [40].

**Table 1:** Primers for real-time PCR used in this study

**Gene target**	**Primer name**	**Sequence (5′-3′)**
TNF-α	TNF-α For	GCAGGTTCCGTCCCTCTCAT
	TNF-α Rev	TGCCAGTTCCACATCTCGGA
IL-1β	IL-1β For	CGTCCTCTGTGACTCGTGGG
	IL-1β Rev	ATGGGTCAGACAGCACGAGG
iNOS	iNOS For	GGGAACACCTGGGGATTTTC
	iNOS Rev	CACAGTTTGGTCTGGCGAAG
Nrf2	Nrf2 For	GGATATTCCCAGCCACGTTGA
	Nrf2 Rev	AATCAGTCATGGCCGTCTCC
HO-1	HO-1 For	GTTTCCTGTTGGCGACCGTG
	HO-1 Rev	GCCAGGCAAGATTCTCCCCT
Gas6	Gas6 For	TCGGATAGCACCTGGATCGT
	Gas6 Rev	ACTGCTGGTGATGCGTCCA
β-actin	β-actin For	GATCAGCAAGCAGGAGTACGATGA
	β-actin Rev	GGTGTAAAACGCAGCTCAGTAACA

### Statistical Analysis

All data represent the results of at least three independent experiments. Data were blindly analyzed using GraphPad Prism Software, version 6.0 (GraphPad Software, Inc., San Diego, CA, USA). Results are expressed as mean ± SD. Data were analyzed by one-way analysis of variance (ANOVA) test followed by Bonferroni’s post hoc test for multiple comparisons. A value of *p* < 0.05 was considered to indicate statistically significant differences. Additional details are provided in the figure legends, where appropriate.

## Results

### Analysis of *Euglena gracilis* Extract

**Fig. 1:**
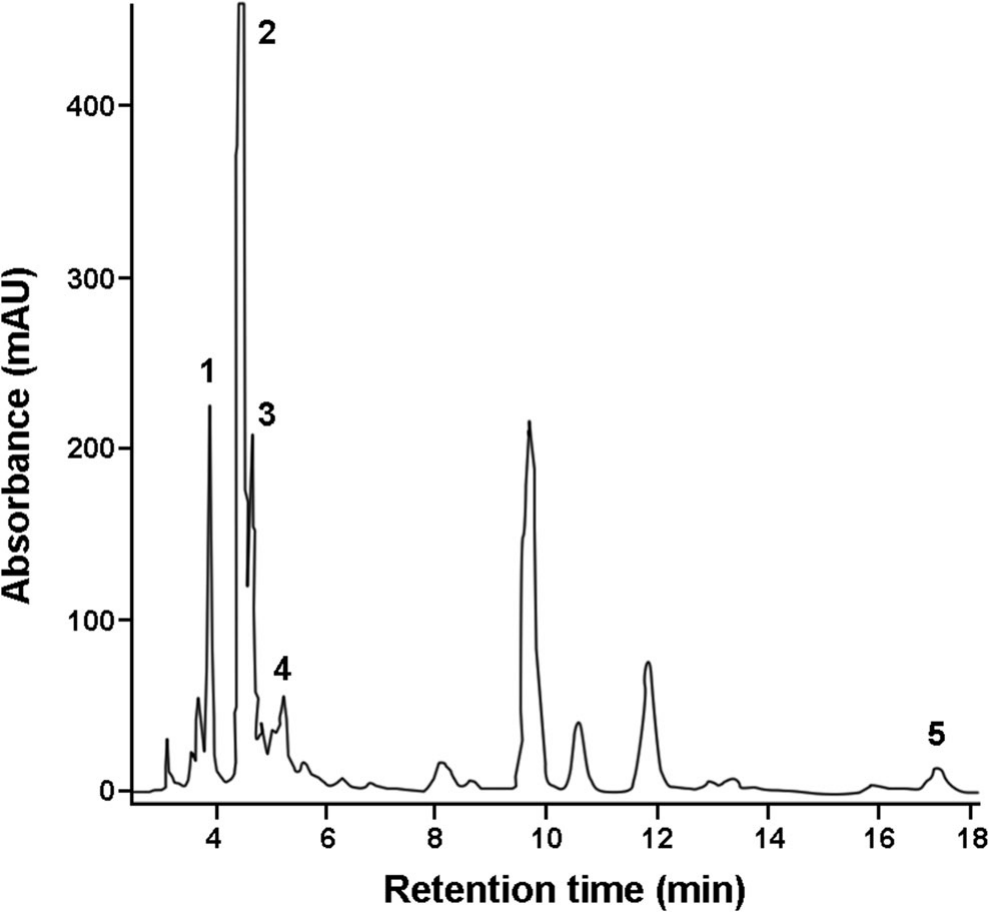
HPLC profile of the Euglena acetone extract. Chromatogram was registered at 460 nm (retention times: neoxanthin 3.8 min (1); diadinoxanthin 4.4 min (2); zeaxanthin 4.65 min (3); canthaxanthin 5.12 min (4); β-carotene 17.7 min (5); unidentified xanthophylls 3.0–3.7, 4.8, 6.5 min; chlorophylls 8–14 min)

Acetone is the preferable solvent for the extraction of a full range of pigments. Considering that it can extract most photosynthetic pigments with a wide range of polarity, acetonic extracts are designed as the richest in carotenoids [41]. The content of carotenoids in EAE was 17.8 mg/g of dry extract. HPLC UV-Vis analysis led to the identification of β-carotene and several xanthophylls (Fig. 1). Table 2 shows the relative content of carotenoids expressed as percentage of the total carotenoid content.

**Table 2:** Relative content of the main carotenoids in *Euglena gracilis* extract

**Carotenoid**	**%**
Neoxanthin	17
Diadinoxanthin	45
Zeaxanthin	14
Canthaxanthin	10
β-carotene	8
Unidentified xanthophylls	6

Xanthophylls account for 92% of the total carotenoids and diadinoxanthin is the most abundant; β carotene represents only 8%

### Determination of Non-cytotoxic Concentrations of *Euglena gracilis* Extract in Microglial Cells

**Fig. 2:**
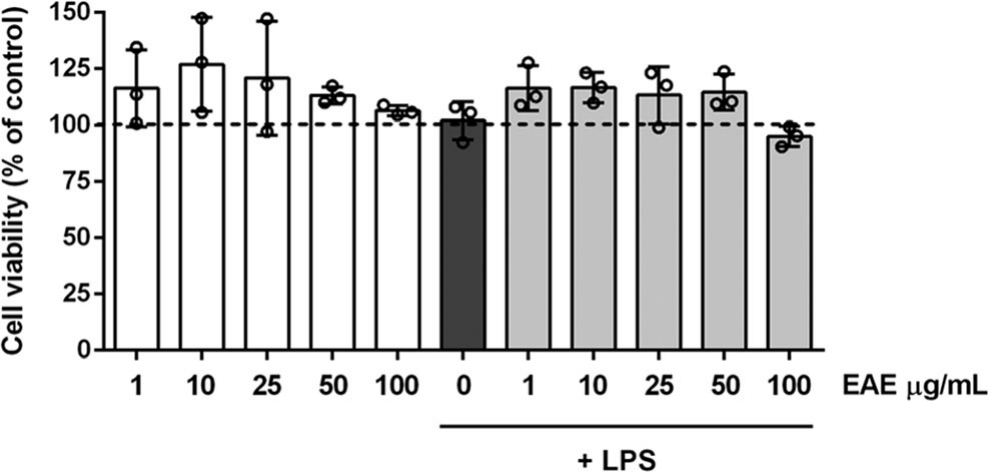
Effects of *Euglena gracilis* extract in microglia cell viability. Microglia were cultured for 24 h in 10% serum-containing medium, which was replaced with serum-free medium before pre-treatment with EAE (1–100 μg/mL) in the absence (white bars) or presence of LPS (gray bars) for 16 h. At the end of incubation, cell viability was determined by SRB assay. Results are expressed as percentage of cell viability relative to control cells. Data are presented as means ± SD (*n* = 3 in triplicate) and there is no significant difference in cell viability. Dashed line indicates vehicle-treated cells

We first performed experiments to examine the safety and identify the non-cytotoxic concentrations of EAE in microglia. Cultures were serum starved for 2 h and then incubated with increasing concentrations of the extract applied alone or in the presence of LPS stimulation for 16 h. EAE did not exhibit any significant effect on microglia survival at concentrations ranging from 1100 μg/mL, either in the absence or presence of LPS (Fig. 2).

### *Euglena gracilis* Extract Reduces Microglia Inflammatory Response to LPS

**Fig. 3:**
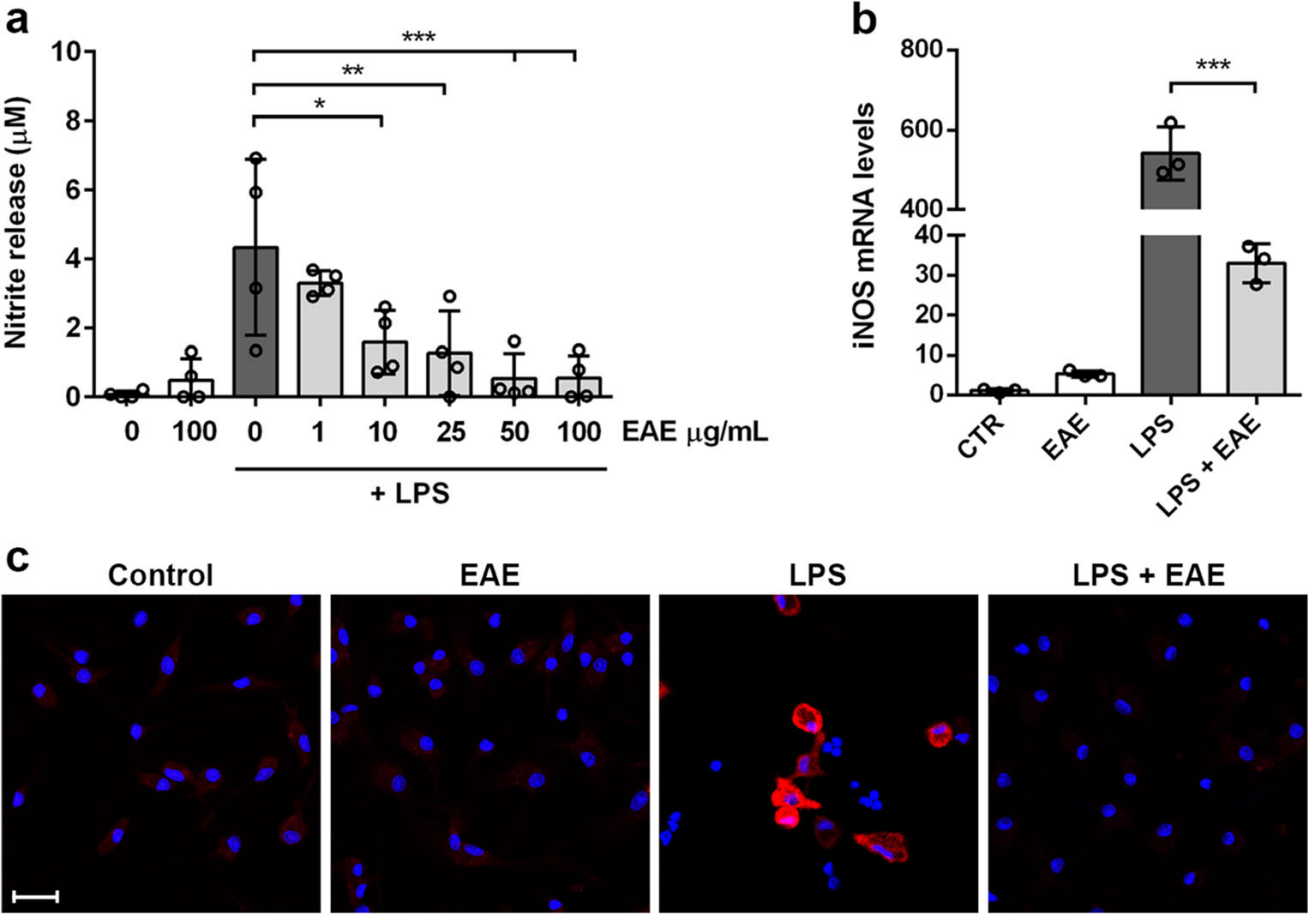
Effects of *Euglena gracilis* extract on nitrite release and iNOS expression in LPS-stimulated cortical microglia. Microglia were cultured for 24 h in 10% serum-containing medium, which was replaced with serum-free medium before pre-treatment with EAE for 1 h followed by stimulation with 100 ng/mL LPS. **a** Nitrite release was measured using Griess reagent. Data are presented as means ± SD (*n* = 4 in triplicate). **b** iNOS mRNA levels were determined by real-time PCR. Data are presented as means ± SD (*n* = 3 in triplicate). **c** Microglia were stained with anti-iNOS antibody (red) and nuclei with DAPI (blue). Experiments were performed 3 times and representative immunofluorescence images are shown. Scale bar, 20 μm. *p < 0.05, **p < 0.01, and ***p < 0.001 versus LPS stimulation (dark gray bars), ANOVA followed by Bonferroni’s post hoc test

Once activated, microglia release pro-inflammatory and neurotoxic factors like ROS, NO, and IL-1β and TNF-α, among other pro-inflammatory cytokines [4]. To test whether EAE could exert anti-inflammatory effects, microglia activation was induced by LPS. Firstly, the release of nitrites, the oxidized metabolites of NO, has been quantified as an index of inflammatory activation. EAE significantly blocked the LPS-induced nitrite release by microglial cultures, starting from the concentration of 10 μg/mL (Fig. 3a). Based on these results, we selected the extract concentration of 100 μg/mL to explore the effect on mRNA and protein levels of iNOS, enzyme generating large amounts of NO in response to LPS, cytokines, or other agents [42]. After LPS stimulation, iNOS was over-expressed, and EAE strongly prevented its gene and protein expression (Fig. 3b and c). In addition, EAE had no effects when incubated alone (Fig. 3).

**Fig. 4:**
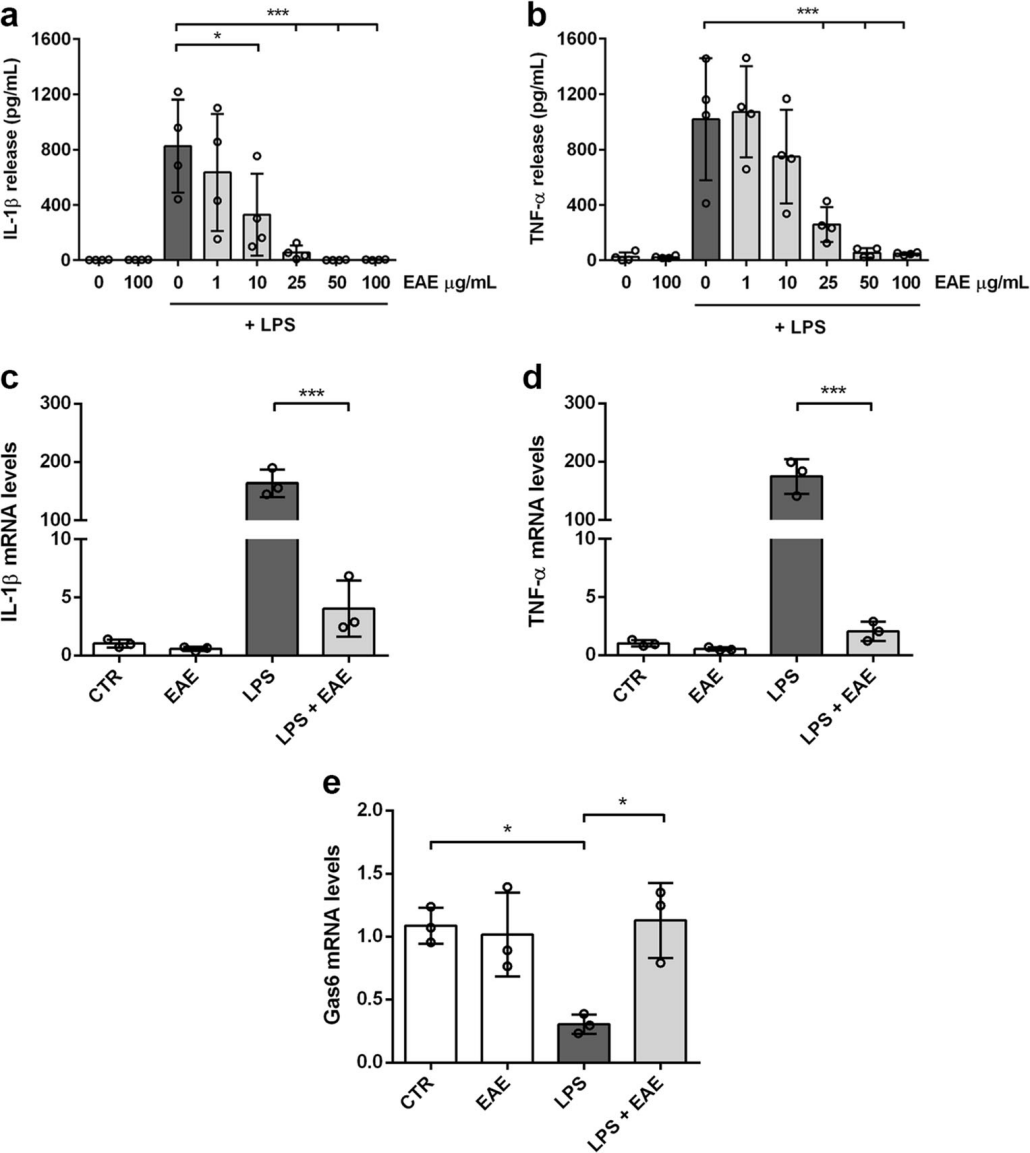
Effects of *Euglena gracilis* extract on cytokine production and release from LPS-stimulated cortical microglia. Microglia were cultured for 24 h in 10% serum-containing medium, which was replaced with serum-free medium before pre-treatment with EAE for 1 h followed by stimulation with 100 ng/mL LPS. Supernatants were collected and analyzed for **a** IL-1β and **b** TNF-α release. Data are means ± SD (*n* = 4 in triplicate). **c** IL-1β, **d** TNF-α, and **e** Gas6 mRNA levels were quantified by real-time PCR. Data presented as means ± SD (*n* = 3 in triplicate). *p < 0.05 and ***p < 0.001 compared to control cells or LPS stimulation (dark gray bars), ANOVA followed by Bonferroni’s post hoc test

The pro-inflammatory phenotype of microglial cells is also characterized by the increased release of IL-1β and TNF-α, two master regulators of inflammation widely implicated in the pathogenesis of CNS disorders, that share the same kinetics of release [43, 44]. Unstimulated cells released low amounts of IL-1β and TNF-α which remained unchanged after treatment with the highest non-cytotoxic concentration of the extract. Conversely, in the presence of LPS, EAE reduced the release of the two pro-inflammatory cytokines in a concentration-dependent manner. In particular, the extract completely inhibited the release of both cytokines starting from the concentration of 25 μg/mL (Fig. 4a and b). Furthermore, treatment of microglia with the highest non-cytotoxic concentration of EAE (100 μg/mL) suppressed the LPS-induced increase of mRNA levels of the two pro-inflammatory cytokines (Fig. 4c and d). The inhibitory activity of Euglena extract on IL-1β and TNF-α expression and release well correlated with the inhibitory activity observed on nitrite production and iNOS expression, confirming the anti-inflammatory properties of the studied extract.

The protective effect of EAE has been further explored by studying its role on microglial phagocytosis, a crucial process required to control CNS homeostasis and diseased states [45]. To this purpose, the effect of EAE on growth arrest–specific gene 6 (Gas6) expression, known to be involved in the phagocytic activity of microglia [46, 47], has been evaluated. EAE pretreatment completely reverted the downregulation of Gas6 induced by LPS (Fig. 4e), suggesting that the effect on phagocytosis could be part of the anti-inflammatory mechanism of EAE.

### *Euglena gracilis* Extract Inhibited NF-κB Signaling in Microglia Cells

**Fig. 5:**
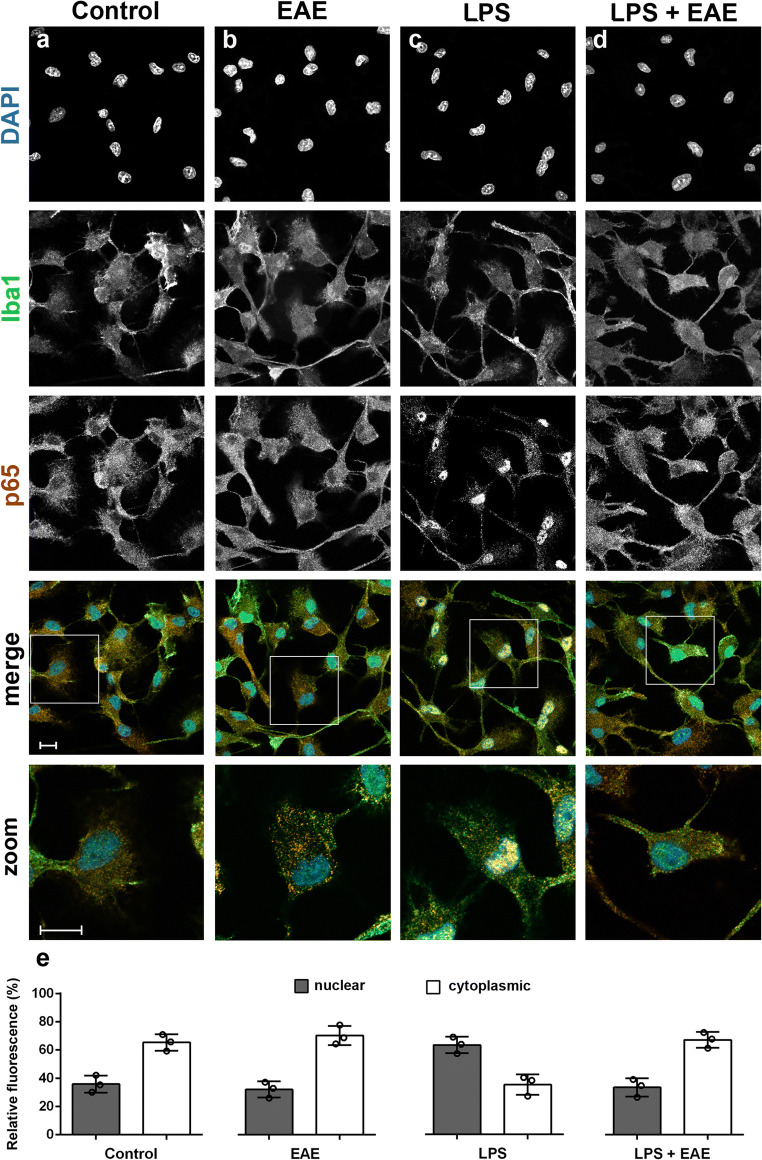
Effects of *Euglena gracilis* extract on NF-κB activation in unstimulated and LPS-stimulated microglia. Cells were subcultured for 24 h in 10% serum-containing medium, which was replaced with serum-free medium before stimulation with 100 μg/mL EAE ± 100 ng/mL LPS. **a–d** Cells were then processed for NF-κB p65 (red) and Iba1 (green) immunostaining. Experiments were performed 3 times and representative confocal images showing subcellular localization of p65 are shown. Scale bar, 10 μm. **e** The fluorescence intensity of cytoplasmic and nuclear p65 subunit was calculated using ImageJ software and results are presented as a percentage of nuclear (dark gray bars) over cytoplasmic NF-κB p65 (white bars). Data are mean ± SD from three independent experiments (*n* = 3)

The transcription factor NF-κB is a key regulator of inflammation activated in response to pro-inflammatory stimuli and results in increased expression of many cytokines and chemokines [48]. Thus, to define whether EAE suppressed microglia inflammatory response acting on NF-κB signaling pathway, we monitored the nuclear translocation of p65 subunit, as an indicator of NF-κB signaling activation. EAE alone did not induce NF-κB activation, as shown by a predominantly cytoplasmic distribution of p65 subunit, similar to that observed in untreated control cells (Fig. 5a, b, and e). After LPS exposure, microglia showed a predominant nuclear p65 immunoreactivity (Fig. 5c and e), that was suppressed by pre-treatment with EAE (Fig. 5d and e). These results suggest that NF-κB signaling participates in the anti-inflammatory effect of EAE.

### *Euglena gracilis* Extract Activates the Nrf2 Pathway in Microglial Cells

**Fig. 6:**
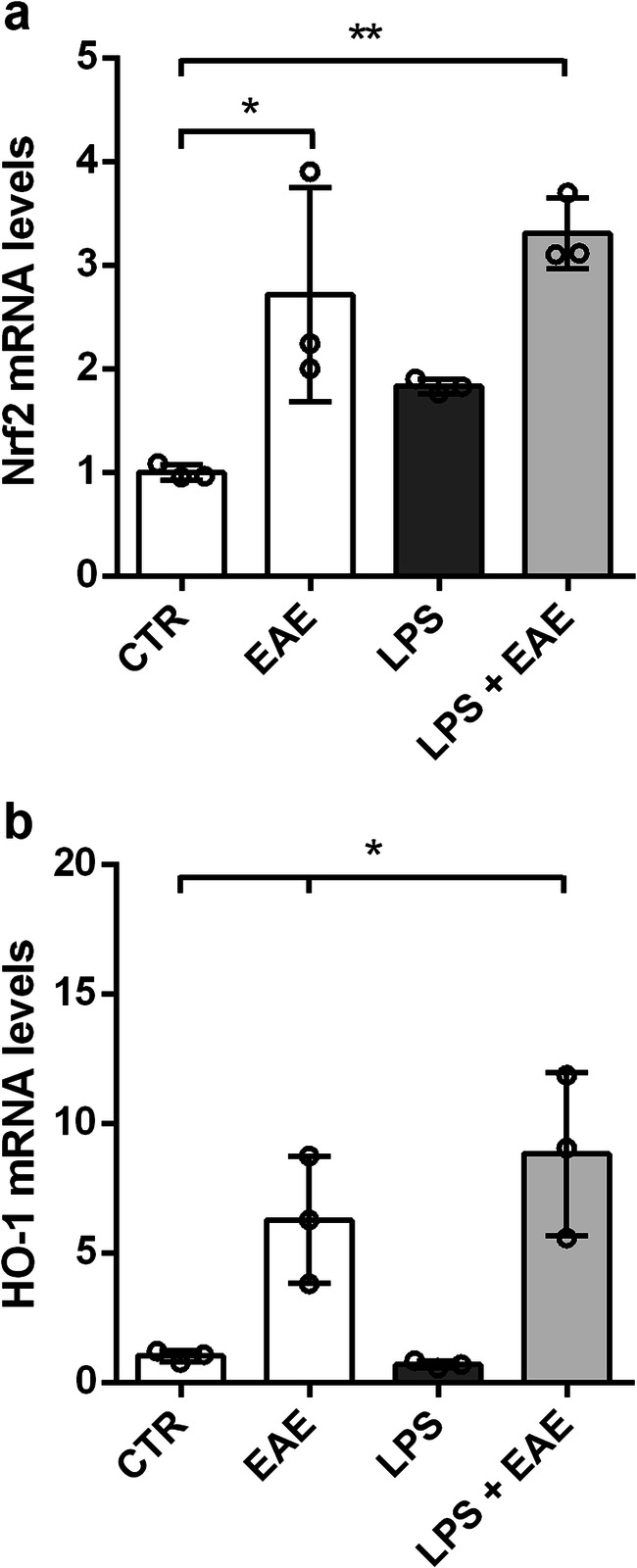
Effects of *Euglena gracilis* extract on Nrf2 signaling in LPS-stimulated cortical microglia. Microglia were cultured for 24 h in 10% serum-containing medium, which was replaced with serum-free medium before pre-treatment with EAE for 1 h followed by stimulation with 100 ng/mL LPS for 6 h. **a** Nrf2 and **b** HO-1 mRNA levels were quantified by real-time PCR. Data are presented as means ± SD (*n* = 3 in triplicate). *p < 0.05 and **p < 0.01 compared to control cells. ANOVA followed by Bonferroni’s post hoc test

The Nrf2 pathway controls the physiological cellular redox homeostasis status, regulating the expression of various antioxidant and detoxifying enzymes, including HO-1 [49]. It is not unreasonable to presume that EAE could reduce microglia inflammatory response via activation of the Nrf2 antioxidant pathway. To test this, we analyzed the effect of EAE on the microglia expression of Nrf2 and one of its target genes, namely HO-1. EAE was able to upregulate the mRNA levels of Nrf2 and HO-1 both in the absence and presence of LPS (Fig. 6), indicating the activation of the Nrf2 pathway.

### The Anti-inflammatory Properties of *Euglena gracilis* Extract Are Nrf2 Independent

**Fig. 7:**
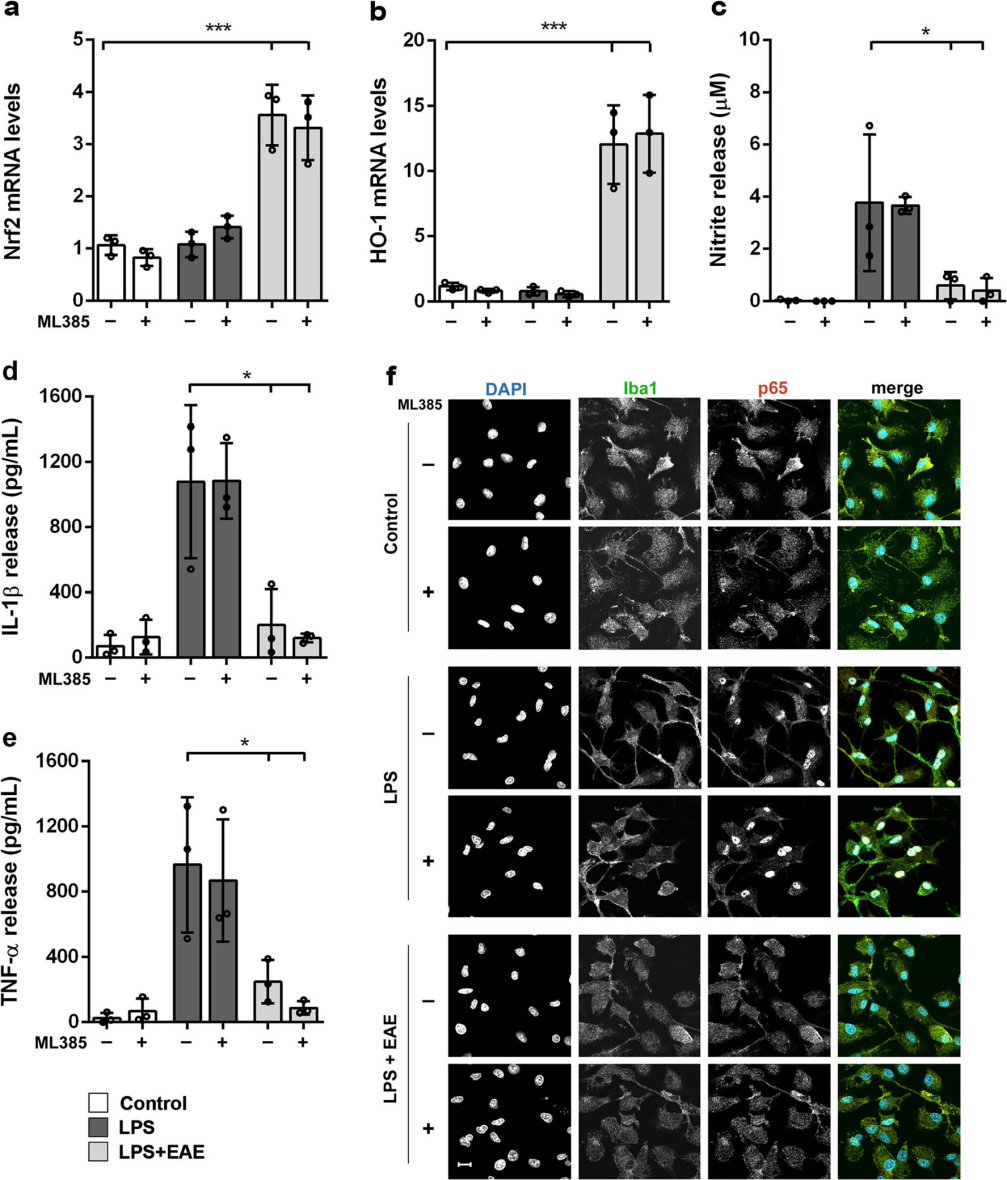
Effect of Nrf2 inhibition on the anti-inflammatory properties of the *Euglena gracilis* extract. Microglia were cultured for 24 h in 10% serum-containing medium, which was replaced with serum-free medium before pre-treatment with ML385 (20 μM) and EAE (25 μg/mL) followed by stimulation with 100 ng/mL LPS. **a** Nrf2 and **b** HO-1 mRNA levels were quantified by real-time PCR. Supernatants were collected and analyzed for **c** NO, **d** IL-1β, and **e** TNF-α release. Data are means ± SD (*n* = 3 in triplicate). *p < 0.05 and ***p < 0.001 compared to control cells (white bars) or LPS stimulation (dark gray bars); ANOVA followed by Bonferroni’s post hoc test. **f** Cells were processed for NF-κB p65 (red) and Iba1 (green) immunostaining. Experiments were performed 3 times and representative confocal images showing subcellular localization of p65 are shown. Scale bar, 10 μm

To assess the relevance of Nrf2 signaling in the anti-inflammatory effect of EAE in LPS-stimulated microglia, ML385, a specific Nrf2 inhibitor [50], was employed. Microglia were pretreated with or without the inhibitor (20 μM) [51], then treated with EAE followed by a further incubation with LPS. Unexpectedly, ML385 did not change the increased mRNA expression levels of Nrf2 and HO-1 induced by EAE (Fig. 7a and b). Furthermore, the EAE-reduced release of nitrites, IL-1β, and TNF-α was not affected by the addition of Nrf2 inhibitor (Fig. 7c–e). Likewise, in the presence of ML385, EAE suppressed NF-κB activation after LPS stimulation to the same extent as did in the absence of Nrf2 inhibitor (Fig. 7f). These results suggest that, although EAE upregulates the expression of Nrf2 and HO-1, it controls microglia inflammatory response by a Nrf2-independent mechanism.

## Discussion

Attenuation of microglia activation and understanding molecular mechanisms involved in this process may be crucial for developing therapeutic interventions to alleviate neuronal injury and effectively treat CNS diseases. In this context, a successful therapeutic strategy is to consider using pharmacological agents that affect at the same time multiple mechanisms that drive microglial activation. Natural products, including microalgae, that target multiple mechanisms and in combination may exert synergistic effects could be a valuable source to provide candidates to be used as a long-term strategy to treat chronic inflammatory CNS diseases [52]. In particular, microalgae are considered very interesting natural sources of compounds with a broad spectrum of biological activities. Indeed, microalgae synthesize vitamins, proteins, soluble fibers, minerals, and pigments with potential health benefits [14–16], including the anti-inflammatory ones [53–57].

The phytochemical profile of the extract of Euglena used in this study showed the presence of several carotenoids, which are the most abundant lipid-soluble phytochemicals with antioxidant, antiapoptotic, and immunomodulatory properties [19, 58]. Furthermore, several clinical studies showed that a high intake of carotenoids (i.e., astaxanthin, lutein, zeaxanthin), from food or supplements, correlates with improvements in cognitive, learning, and memory functions. These effects are supposed to be due to the antioxidant action of carotenoids and the resulting positive effect on neuronal membrane integrity [59–61]. Based on these results and considering that carotenoids cross the blood-brain barrier [62], we investigated whether the carotenoid-rich extract from the microalga Euglena could exert anti-inflammatory activity in microglia cultures. When LPS-stimulated microglia were exposed to non-cytotoxic concentrations of EAE, cells showed a suppressed expression of iNOS and NO release. NO is a versatile molecule particularly important in the CNS, where it participates in synaptic plasticity, neurotransmitter release, and also in immune response. However, when produced in excess via iNOS by activated microglia, NO contributes to acute and chronic inflammation and causes neuronal cell death, directly or by its oxidation products, such as peroxynitrite, which is produced by the oxidation of NO with superoxide [63–67]. Elevated levels of pro-inflammatory cytokines, such as TNF-α, IFN-γ, IL-1β, IL-6, and IL-18, have also been shown in various inflammatory conditions and, in this regard, a recent study showed that the microalga *Euglena tuba* can counteract the increased levels of inflammatory cytokines in an LPS-induced model of systemic inflammation [27]. Here we showed that in LPS-induced microglia activation, the carotenoid-enriched extract significantly reduced gene expression and release of IL-1β and TNF-α, confirming its anti-inflammatory effect. These results confirm previous studies that showed the ability of carotenoids, such as β-carotene, lycopene, astaxanthin, and lutein, to reduce the expression of CNS inflammatory markers, supporting the hypothesis that the therapeutic potential of carotenoids in various neurodegenerative diseases can be mediated by their anti-neuroinflammatory effects [19, 58, 68]. Furthermore, treatment of microglia with EAE completely reverted the LPS-induced downregulation of Gas6, a ligand of TAM receptors, known to promote phagocytosis in microglia [46], suggesting that the anti-inflammatory effect of the extract could be associated with the beneficial effect on microglia phagocytic activity.

Despite the large number of studies on anti-inflammatory effects of carotenoids, the underlying mechanisms remain unclear. Several natural products exert anti-inflammatory effects by influencing NF-*κ*B signaling, able to modulate the expression of genes for inflammatory cytokines [69–72]. In particular, mechanistic in vitro studies have shown that carotenoids can reduce NF-κB activation by binding to the kinase responsible for the phosphorylation of IκBα, blocking its ubiquitylation and dissociation, and preventing the translocation of p65 subunit to the nucleus, preventing the downstream production of inflammatory cytokines [58, 73]. In microglia cells, NF-κB p65 nuclear translocation induced by LPS was significantly attenuated by pre-treatment with the extract of Euglena. Therefore, inhibition of the activation of the NF-κB signaling pathway could be one of the potential anti-inflammatory mechanisms of EAE.

Carotenoids can affect other cellular signaling cascades, such as mitogen-activated protein kinases or the transcription factor Nrf2 [58]. Under injurious conditions, the latter regulates the expression of antioxidant, anti-inflammatory, and cytoprotective genes to restore redox homeostasis [74]. Furthermore, HO-1, a target gene of the Nrf2 pathway, is endowed with anti-inflammatory effects, and its enzymatic metabolites, such as carbon monoxide, biliverdin, and Fe^2+^, can inhibit the activation of NF-κB [75]. Therefore, we speculated that the activation of the Nrf2/HO-1 signaling pathway could contribute to the mechanism of the anti-inflammatory action of EAE. In our experimental conditions, the extract per se increased gene expression of Nrf2 as well as its downstream gene HO-1, indicating Nrf2 signaling activation. These results suggest that Nrf2 signaling may be part of the mechanism underlying the anti-inflammatory effect of the studied extract. However, the Nrf2 inhibitor ML385 had no effect either on the increased expression of Nrf2 and HO-1 or on the reduced release of NO, IL-1β, and TNF-α induced by EAE. Therefore, the extract could exert anti-inflammatory actions in a Nrf2-independent manner. Furthermore, pharmacological inhibition of Nrf2 did not affect either the inhibitory effect of EAE on nuclear translocation of the NF-κB p65 subunit, suggesting that the extract action on NF-κB signaling is Nrf2 independent. Latter results are in contrast with previous studies showing that, Nrf2 inhibition or deficiency leads to more severe inflammation [76]. However, our results are consistent with other studies that have shown Nrf2-independent anti-inflammatory properties of several Nrf2 inducers [77–81].

## Conclusions

The results of this study indicate that an acetone extract of the microalga *Euglena gracilis*, rich in carotenoids, exerts anti-inflammatory activities in an in vitro model of neuroinflammation. Moreover, we identified two molecular mechanisms underlying the anti-inflammatory effect of this extract that involve the inhibition of NF-κB and the activation of Nrf2 signaling pathways. However, although both pathways are regulated by EAE, they might contribute to the anti-inflammatory effect of the extract in an independent manner. Furthermore, other potential cellular mechanisms must be considered. As an example, modulation of histone deacetylases, which play a critical role in the regulation of inflammation [82], could be taken into account as an additional anti-inflammatory mechanism of EAE. Even so, these findings can provide the pharmacological foundation for further studies aimed at testing single carotenoids of the extract to identify which constituent(s) is/are responsible for the anti-neuroinflammatory effect. These studies will clarify whether the anti-inflammatory effect of EAE could be due to a single carotenoid or represent a synergistic effect of combined carotenoids of the extract, as the result of regulating multiple pathways. In addition, in vivo studies will be necessary to confirm these observations and to investigate the ability of EAE constituents to cross the blood-brain barrier, even though most carotenoids are lipophilic and able to reach the brain in biologically relevant concentrations [62].

## Data Availability

The datasets generated and/or analyzed during the current study are available from the corresponding author on reasonable request.
